# Coupling Drug Dissolution with BCS

**DOI:** 10.1007/s11095-024-03661-x

**Published:** 2024-01-30

**Authors:** Antony Simitopoulos, Athanasios Tsekouras, Panos Macheras

**Affiliations:** 1 Faculty of Pharmacy, National and Kapodistrian University of Athens, Athens, Greece; 2 Department of Chemistry, National and Kapodistrian University of Athens, Athens, Greece; 3 PharmaInformatics Unit, ATHENA Research Center, Athens, Greece

**Keywords:** BCS, dissolution, drug classification, finite dissolution time, mean dissolution time

## Abstract

**Purpose:**

The purpose of this study is to develop a Temporal Biopharmaceutic Classification System (T-BCS), linking Finite Dissolution Time (F.D.T.) and Mean Dissolution Time (M.D.T.) for Class I/III drugs and Mean Dissolution Time for saturation (M.D.T.s.) for Class II/IV drugs.

**Methods:**

These parameters are estimated graphically or by fitting dissolution models to experimental data and coupled with the dose-to-solubility ratio (*q*) for each drug normalized in terms of the actual volume of dissolution medium (900 mL).

**Results:**

Class I/III drugs consistently exhibited *q* values less than 1, aligning with expectations based on their solubility, while some Class II/IV drugs presented a deviation from anticipated *q* values, with observations of *q* < 1. This irregularity was rendered to the dissolution volume of 250 mL used for biopharmaceutical classification purposes instead of 900 mL applied as well as the dual classification of some sparingly soluble drugs. Biowaivers were also analyzed in terms of M.D.T., F.D.T. estimates and the regulatory dissolution time limits for rapidly and very-rapidly dissolved drugs.

**Conclusions:**

The T-BCS is useful for establishing correlations and assessing the magnitude of M.D.T., F.D.T., or M.D.T.s. for inter- and intra-class comparisons of different drugs and provide relationships between these parameters across all the models that were utilized.

**Graphical Abstract:**

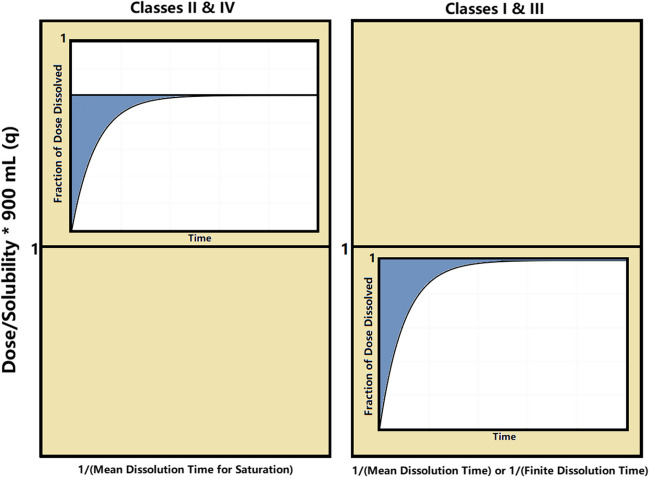

## Introduction

In a recent article [1] we introduced the concept of Finite Dissolution Time (F.D.T.) as an intuitive extrapolation of Finite Absorption Time (F.A.T.) concept [2]. This is a plausible extrapolation since drug dissolution takes place under *in viv*o conditions for a finite time regardless of the complete or incomplete dissolution of the dose administered. The finite character of both terms, F.A.T.and F.D.T. is physiologically sound since drug absorption does not take place beyond the absorptive sites while drug dissolution is also not important beyond the absoprtive sites. Accoringly, the inception of the F.A.T. and F.D.T. concepts are linked with the physiological constraints of the dissolution and absorption processes under *in vivo* conditions. However, it is not uncommon to see *in vitro* dissolution profiles reaching a plateau value of 100% of dose dissolved at finite time [3, 4]. In this particular case, the term F.D.T. denotes the time needed for the complete dissoution of the drug dose. Besides, the F.D.T. is, in essence, included in the today’s regulatory biowaiver guidelines [5, 6]; this is so since the rapid (< 30 min) or very rapid (< 15 min) dissolution criterion for the biowaivers imply completion of the dissolution process in finite time. Intuitively, the F.D.T estimate, *τ*_d_, considered under *in vivo* conditions is equal or shorter than the F.A.T. estimate *τ*_,_ namely, *τ*_d_ ≤ *τ* [1, 2, 7]. For Class II drugs, *τ*_d_ = *τ*, for Class I and III drugs *τ*_d_ < *τ* while for Class IV drugs both relationships i.e., *τ*_d_ = *τ* and *τ*_d_ < *τ* are possible [7]. We should note that a Class II drug with basic properties can be completely dissolved in the stomach, not precipaitate and be absorbed in the intestine and essentially bahave like BCS Class I drug.

So far, the mean dissolution behavior of solid drug particles has been quantified with the mean dissolution time (M.D.T.) and the mean dissolution time for saturation (M.D.T.s.) for drugs whose dose is completely or not completely dissolved at the end of the dissolution process, respectively [8]. Both terms correspond to a stochastic interpretation of the dissolution process since the profile of the accumualtaed fraction of drug amount dissolved from a solid dosage form gives the probability of the residence times of the drug molecules in the dissolution medium. The fraction of drug dissolved is always a distribution function, and therefore it can be characterized by its first (statistical) moment, which is the M.D.T. The latter term holds only when the entire available drug dose is dissolved completely. When drug particles remain undissolved at the end of the dissolution process, the M.D.T. is not defined since it is equal to infinity. In this case, the Mean Dissolution Time for saturation (M.D.T.s.) is coined and refers only to the portion of the dose that is actually dissolved [8]. Unfortunately, the clear distinction between (M.D.T.) and (M.D.T.s.) has neither been recognized nor adopted in the literature so far. In this work, we show that the three parameters, (F.D.T.), (M.D.T.) and (M.D.T.s.), lie in the heart of the biopharmaceutic classification system (BCS) [9]; this allowed us to couple dissolution time considerations with BCS. A dissolution-based temporal version of BCS, the so-called T- BCS was developed.

## Theory

The temporal classification of Class I and III drugs whose dose is completely dissolved in the dissolution medium are based on the M.D.T. values, while Class II and IV drugs whose dose is not completely dissolved in the dissolution medium are classified according to their M.D.T.s. values using the time axis (M.D.T.)^−1^ or (M.D.T.s.)^−1^, respectively. In addition, drugs/formulations which exhibit finite dissolution time (F.D.T.) for complete dissolution of the drug dose can be also classified in Class I or III using the (F.D.T.)^−1^ axis. The dimensionless dose/solubility ratio, *q,* normalized in terms of the volume (900 mL) of dissolution medium, Eq. 1 [8, 9].

1 $$q=\frac{Dose}{{C}_{s} V}$$

### Graphical estimation of M.D.T. or M.D.T.s.

**Fig. 1:**
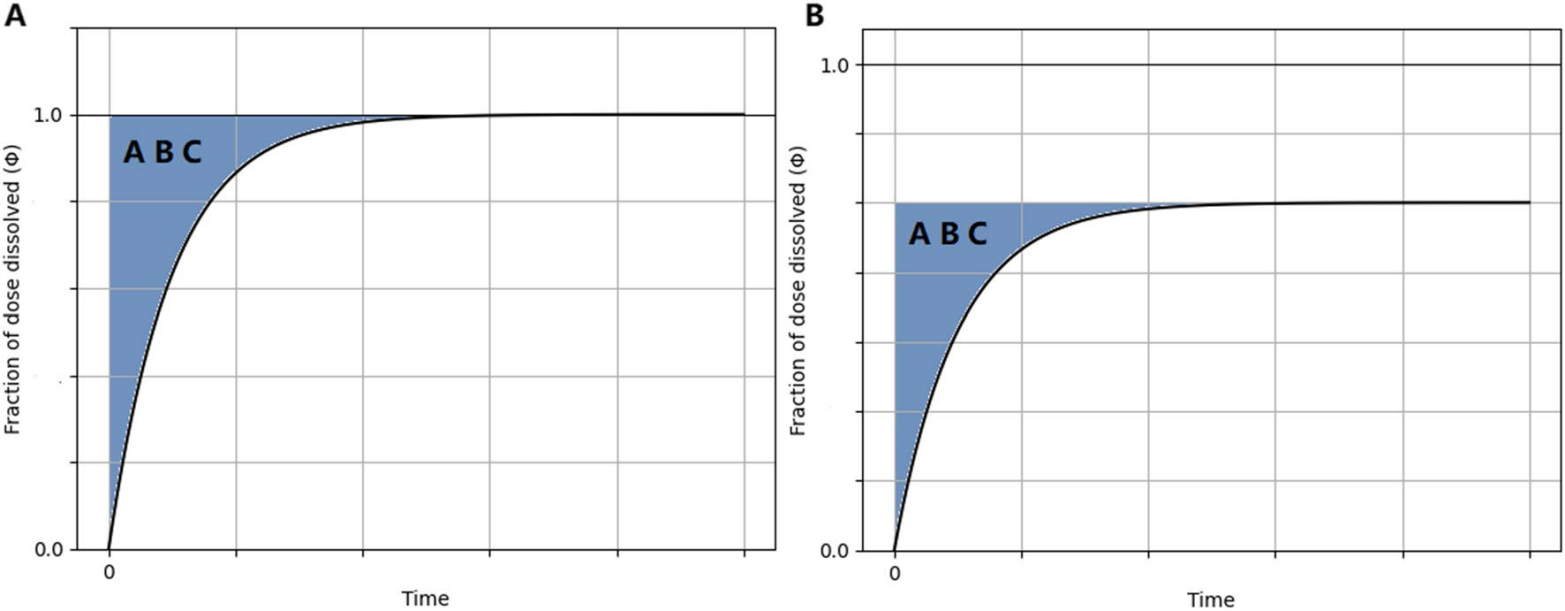
Graphical estimation of M.D.T. (**A**) and M.D.T.s. (**B**) from the experimental fraction of dose dissolved (Φ)-time data. The shaded region denotes the Area Between the Curves (ABC).

The mean dissolution time (M.D.T.) corresponds to the first moment that can be determined from the experimental dissolution data using the following equation [8]:

2 $$M.D.T.=\frac{{\int }_{0}^{{W}_{\infty }}t*dW(t)}{{\int }_{0}^{{W}_{\infty }}dW(t)}$$

where *W*(*t*) is the cumulative amount of drug dissolved at time *t*. Estimates for M.D.T. or M.D.T.s. can be obtained graphically by calculating the area (ABC) between the fraction of dose dissolved (*Φ*) – time curve and the plateau level, Fig. 1. When the plateau level is equal to one (*Φ*_*∞*_ = 1) an estimate for M.D.T. can be derived from Eq. 3, Fig. 1a. Similarly, an estimate for M.D.T.s. can be derived from Eq. 3 when the plateau level is *Φ*_*∞*_ < 1, Fig. 1b.

3 $$M.D.T.\;or\;M.D.T.s.=\frac{ABC}{{\Phi }_{\infty }}$$

### The Noyes-Whitney Equation Model

Since the very first experiment in 1897, dissolution is mathematically described by the Noyes-Whitney equation [10–12]; the integrated form of the dissolved drug concentration *C,* as a function of time *t* indicates that the dissolution profile is exponentially reaching the plateau value, the saturation solubility* C*_s_ at infinite time, Eq. 2

4 $$C={C}_{{\mathrm{s}}}[1-{e}^{-kt}]$$

where *k* is the dissolution rate constant. This equation can be expressed as a function of the fraction of dose dissolved *Φ* when *q* ≥ 1 as follows,

5 $$\Phi =\frac{1}{q}[1-{e}^{-kt}]$$

which means that only a portion of the dose is dissolved, and the drug reaches the saturation level 1/q [13]. In this case, the corresponding M.D.T.s. is equal to *1/k* [8, 14]. On the contrary, when *q* < 1*,* which means that the entire dose is eventually dissolved, the dissolution follows the usual exponential form only until it reaches the value *Φ* = 1, i.e., 100% of the drug is dissolved, in a finite dissolution time*, τ*_d_ and thereafter remains constant [13],

6 $$\Phi =\left\{\begin{array}{cc}\frac{1}{q}\left[1-{e}^{-kt}\right],& {\mathrm{for}}\;t<{\tau }_{{\mathrm{d}}}\\ 1,& {\mathrm{for}}\;t\ge {\tau }_{{\mathrm{d}}}\end{array}\right.$$

where:

7 $${\tau }_{{\mathrm{d}}}=-\frac{{\mathrm{ln}}(1-q)}{k}$$

In this case (*q* < 1), the M.D.T. is as follows [8, 13],

8 $$M.D.T.=\frac{q-\left(q-1\right)-{\mathrm{ln}}(1-q)}{kq}$$

which for *q* = 1, i.e., when the dose is equal to the drug amount required to saturate the dissolution medium, collapses to M.D.T. = 1/*k* [8, 13].

### The Weibull Function Model

The Noyes-Whitney equation is distinguished by the assertion that a constant, denoted as the dissolution rate constant *k*, governs the dissolution rate throughout the process. This foundational premise has faced scrutiny in the literature, leading to the emergence of models featuring time-dependent rate coefficients, which are considered to have greater physical relevance with the time-dependent phenomena that occur as dissolution progresses [14]. In this vein, similar analysis has been published [13] for the Weibull function, which is used extensively for the kinetic description of drug dissolution and release data [15, 16]. Therefore, by replacing the dissolution rate constant, *k*, with a time-dependent coefficient, namely, $k={k}_{1}{t}^{-h}$ in the differential Noyes-Whitney equation expressed in terms of *Φ*, we end up with [13]:

9 $$\frac{d\Phi }{dt}={k}_{1}{t}^{-h}\left(\frac{1}{q}-\Phi \right)$$

where *k*_1_ is a constant with time^*h*−1^ units and *h* is a dimensionless constant. Solving Eq. 9 and replacing $a=\frac{{k}_{1}}{1-{\mathrm{h}}}\;and\;b=1-h$, we get a function of the fraction of dose dissolved *Φ* when *q* ≥ 1:

10 $$\Phi =\frac{1}{q}\left(1-{e}^{-a{t}^{b}}\right)$$

which also means that only a portion of the dose is dissolved, and the drug reaches the saturation level 1/q [13]. The corresponding M.D.T.s. is equal to:

11 $${M.D.T.s.=a}^{- \frac{1}{ b}} \Gamma \left(\frac{1}{\beta }-1\right)$$

Where Γ(·) is the complete and Γ(·,·) is the incomplete gamma function.

When *q* < 1, the solution takes a branched form as follows [13]:

12 $$\Phi =\left\{\begin{array}{cc}\frac{1}{q}\left(1-{e}^{-a{t}^{b}}\right),& {\mathrm{for}}\;t<{\tau }_{{\mathrm{d}}}\\ 1,& {\mathrm{for}}\;t\ge {\tau }_{{\mathrm{d}}}\end{array}\right.$$

where:

13 $${\tau }_{{\mathrm{d}}}={\left(-\frac{{\mathrm{ln}}\left(1-q\right)}{a}\right)}^{\left(\frac{1}{b}\right)}$$

In this case (*q* < 1), where 100% of the initial dose is dissolved, the M.D.T. is given by [13]:

14 $$M.D.T.=\frac{1}{bq{a}^{{~}^{1}\!\left/ \!{~}_{b}\right.}}\left[b\left(q-1\right)*\left(-{\mathrm{ln}}\left(1-q\right){)}^{{~}^{1}\!\left/ \!{~}_{b}\right.}\right)-\Gamma \left(\frac{1}{b},-{\mathrm{ln}}\left(1-q\right)\right)+\Gamma \left(\frac{1}{b}\right)\right]$$

which for *q* = 1 turns into:

15 $${\mathrm{M}}.{\mathrm{D}}.{\mathrm{T}}. = {a}^{- \frac{1}{b}} \Gamma \left(\frac{1}{b}-1\right)$$

All parameters, *τ*_d_, M.D.T. and M.D.T.s., for the two cases with *q* < 1 and *q* ≥ 1, respectively, derived [14] for the Noyes-Whitney equation and the Weibull function are listed in Table I.

**Table I:** The M.D.T., M.D.T.s. and *τ*_d_ parameters of the Noyes-Whitney equation and the Weibull function for *q* < 1 and *q* ≥ 1

Function (integrated form)	M.D.T (q < 1)	M.D.T (q = 1)	M.D.T.s	${\uptau }_{{\mathrm{d}}}$
Noyes – Whitney $$\Phi =\left\{\begin{array}{cc}\frac{1}{{\mathrm{q}}}\left(1-{{\mathrm{e}}}^{-{\mathrm{kt}}}\right),& {\mathrm{q}}\ge 1,\mathrm{ for\;t}<{\uptau }_{{\mathrm{d}}}\\ 1,& {\mathrm{q}}<1,\mathrm{ for\;t}\ge {\uptau }_{{\mathrm{d}}}\end{array}\right.$$	$\frac{q-\left(q-1\right)ln(1-q)}{kq}$	$\frac{1}{k}$	$\frac{1}{k}$	$-\frac{\mathit{ln}(1-q)}{k}$
Weibull $$\Phi =\left\{\begin{array}{cc}\frac{1}{{\mathrm{q}}}\left(1-{{\mathrm{e}}}^{{-{\mathrm{at}}}^{{\mathrm{b}}}}\right),& {\mathrm{q}}\ge 1,\mathrm{ for\;t}<{\uptau }_{{\mathrm{d}}}\\ 1,& {\mathrm{q}}<1,\mathrm{ for\;t}\ge {\uptau }_{{\mathrm{d}}}\end{array}\right.$$	$\frac{b\left(q-1\right)*\left({-\mathit{ln}\left(1-q\right)}^{{~}^{1}\!\left/ \!{~}_{b}\right.}\right)-\Gamma \left(\frac{1}{b},-\mathit{ln}\left(1-q\right)\right)+\Gamma \left(\frac{1}{\beta }\right)}{bq{a}^{{~}^{1}\!\left/ \!{~}_{b}\right.}}$	${a}^{- \frac{1}{b}} \Gamma \left(\frac{1}{\beta }-1\right)$	${a}^{- \frac{1}{ b}} \Gamma \left(\frac{1}{\beta }-1\right)$	${\left(-\frac{\mathit{ln}\left(1-q\right)}{a}\right)}^{\left(\frac{1}{b}\right)}$

### The Reaction-Limited Dissolution Model

The reaction limited model [17], which relies on a bidirectional chemical reaction involving the undissolved drug species, the freely available solvent molecules, and the resulting drug-solvent complex was also used for the computational work. It's important to note that this study's foundation relies upon two earlier studies, i.e., conducted by Dokoumetzidis and Macheras in 1997 [18] and by Lansky and Weiss in 1999 [19]. The fundamental differential equation expression describing the rate of the dissolution process, is as follows [17]:

16 $$\frac{dC}{dt}={k}_{1}^{*}{\left(\frac{D}{V}-C\right)}^{\lambda }-{k}_{-1}C$$

where *k*_1_* = *k*_1_^*'*^[molecular weight]^(1–*λ)*^* (k*_1_^*'*^ = *k*_1_[*w*_0_]^*b*^, where [*w*_0_] is the initial concentration of the free species), *D* is the initial quantity (dose) in mass units and *λ* is a dimensionless constant. Equations 17–19 provide the mathematical foundation for understanding drug dissolution under various conditions, encompassing scenarios with both homogeneous (*λ* = 1) (Eq. 17) and solvent-abundant (*λ* ≠ 1) conditions (Eq. 18–19) [17]:

17 $$\Phi =\frac{1}{{q}_{ss}}\left(1-{e}^{\left(-\left({k}_{1}^{*}+{k}_{-1}\right)t\right)}\right)$$

18 $$\frac{dC}{dt}={k}_{1}^{*}{\left(\frac{D}{V}-C\right)}^{\lambda }$$

19 $$C=\frac{D}{V}-{\left[{\left(\frac{D}{V}\right)}^{1-\lambda }-\left(1-\lambda \right){k}_{1}^{*}t\right]}^{1/\left(1-\lambda \right)}$$

Equation 19 has the form of a power-law and can be fitted to experimental dissolution data. Unlike the Noyes-Whitney and Weibull models, a formula for the M.D.T. and M.D.T.s. can not be derived, and can only be computed through numerical methods for both *λ* = 1 and *λ* ≠ 1 cases [17]. Consequently, a numerical calculation for the M.D.T. and M.D.T.s., employing Eq. 2 at its basis, was the sole method used for estimation of these parameters.

## Methods

Dissolution profiles of biowaivers, Class I, II, III, and IV drugs were extracted from their respective literature monographs, articles and subsequently digitized to facilitate analysis. Our analytical focus encompassed three distinctive metrics: F.D.T. (*τ*_d_) and M.D.T. for Class I and III drugs and M.D.T.s. for Class II and IV drugs. These metrics were computed through four distinct methods: one involving graphical analysis employing the trapezoidal rule, another employing the Noyes-Whitney equation, a third utilizing the Weibull function and a fourth one utilizing the reaction-limited model of dissolution.

**Fig. 2:**
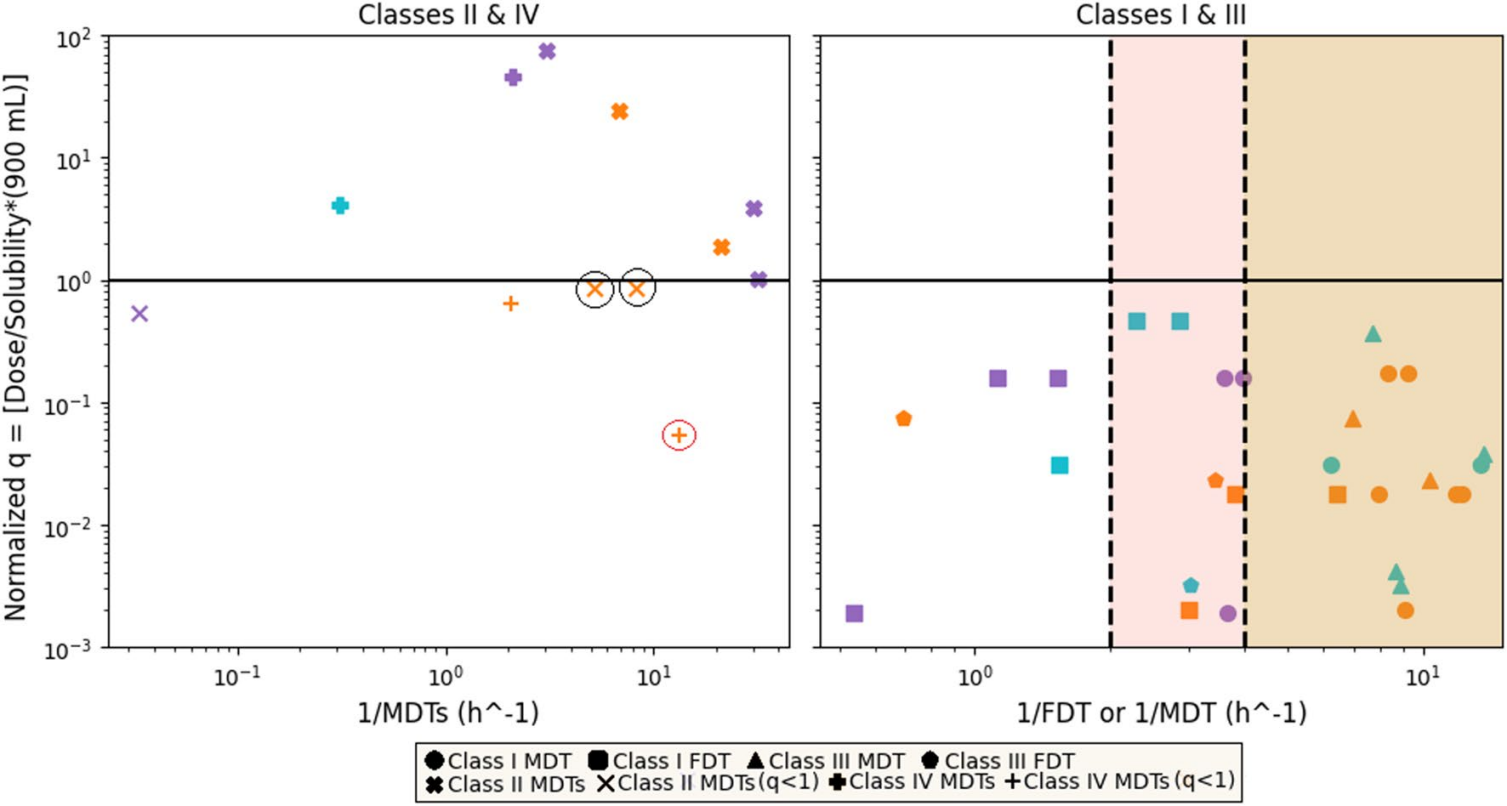
Temporal Biopharmaceutics Classification System (T-BCS) with scattered experimental data from the drugs listed in Table II. For both subplots, the ordinate axis indicates the normalized dose/solubility ratio, *q*, in term of the volume of dissolution medium (900 mL), corresponding to the lowest solubility. On the left subplot, the horizontal time axis (h^−1^, logarithmic scale) corresponds to (1/M.D.T.s.) values for Class II and IV drugs. On the right subplot, the horizontal time axis (h^−1^, logarithmic scale) corresponds to (1/M.D.T.) or (1/F.D.T.) values for Class I and III drugs. The shaded regions indicate the areas for drugs exhibiting rapid (30 min, pink shade at the 2 h^−1^ mark) or very rapid (15 min, beige shade marked at the 4 h^−1^ mark) dissolution in terms of the biowaiver’s criteria. Orange data points correspond to dissolution medium of pH 1.2, purple data points correspond to dissolution medium of pH 4.5 and blue data points correspond to dissolution medium of pH 6.8. See comments about the encircled data points in the discussion.

For the graphical analysis, the computational methodology was inaugurated by a graphical approach to ascertain the F.D.T. metric. A plot depicting % dissolved against time was crafted to articulate the dissolution profile. Essential to this process was the identification of two critical time junctures: the first where % dissolved indicated a value below 100%, succeeded by the subsequent point at which % dissolved surpassed the 100% threshold. A judicious application of linear interpolation was then employed to deduce the precise temporal instant at which % dissolved attained complete dissolution, thereby characterizing the F.D.T. Similarly, for the calculation of the M.D.T. and M.D.T.s., utilizing the dissolution profiles, we assessed the area (ABC) bounded by the dissolution curve and a line parallel to the time-axis aligned with the plateau, Fig. 2. This area (ABC) was subsequently divided by the % dissolved magnitude corresponding to the plateau, Eq. 3, thus yielding the M.D.T. for Classes I and III, as well as the M.D.T.s. for Classes II and IV. Since BCS is based on the minimum solubility across the physiological pH range, for each compound the lowest solubility at its corresponding pH was utilized in the calculations.

For the remaining three models, we systematically performed curve fitting procedures to analyze the experimental data. We employed the equations specified in Table I for the Noyes-Witney and Weibull models and utilized Eq. 19 for the reaction-limited model of dissolution. These curve-fitting analyses were executed within the Python programming environment, particularly employing the SciPy library. The outcome of the curve fittings provided us with parameter estimates, which were subsequently utilized to calculate the M.D.T., M.D.T.s., and F.D.T. (*τ*_d_). In contrast, for the reaction-limited model, explicit expressions for these parameters were unavailable, and as a result, we resorted to numerical methods for the computation of M.D.T. and M.D.T.s. Specifically, for the numerical computation for the reaction-limited model time parameters, Eq. 19 was adapted by incorporating the *D/V* (dose of drug/volume of dissolution medium (900 mL) ratio of each drug to generate the corresponding *W*(*t*) curve, which illustrates the cumulative amount of dissolved drug over time. This curve was subsequently integrated according to Eq. 2 to determine the M.D.T. and M.D.T.s. values, using the corresponding parameter estimates derived from the curve fittings.

## Results

The drugs/drug products are listed in Table II alongside with their BCS classification according to various literature references [20–56].

**Table II:** BCS classification of drugs used in our dissolution analysis

Drug name-Product	BCS Classification
Moxifloxacin Hydrochloride—(Avelox/Moxiflox)	Class I
Zidovudine (Azidothymidine)—(Virex/Lazid/Combivir)	Class I
Sitagliptin Phosphate Monohydrate	Class I
Stavudine	Class I
Primaquine Phosphate	Class I
Amoxicillin Trihydrate—(Innovator/Generic A)	Class I/IV^*^
Lamivudine—(Epivir/Aspen)	Class I/III^*^
Enalapril Maleate—(Vasotec)	Class III
Proguanil Hydrochloride—(Paludrine)	Class III
Metformin Hydrochloride	Class III
Acyclovir—(Zovirax)	Class III/IV^*^
Cimetidine—(Tagamet)	Class III
Paracetamol—(Panadol Extra)	Class I/III^*^
Metoclopramide	Class III
Ketoprofen—(Test/Reference Product)	Class II
Efavirez—(Sustiva)	Class II/IV^*^
Rifampicin	Class II
Ibuprofen	Class I/II^*^
Nifedipine	Class I/II^*^
Piroxicam	Class I/II^*^
Carbamazepine	Class II
Amodiaquine Hydrochloride	Class III/IV^*^
Ciprofloxacin Hydrochloride	Class II/IV^*^
Folic Acid	Class II/IV^*^
Furosemide	Class III/IV^*^

The categorization primarily relies on their respective monograph classifications as documented in the literature. Drugs marked with an asterisk (*) signify dual classifications [57]

**Fig. 3:**
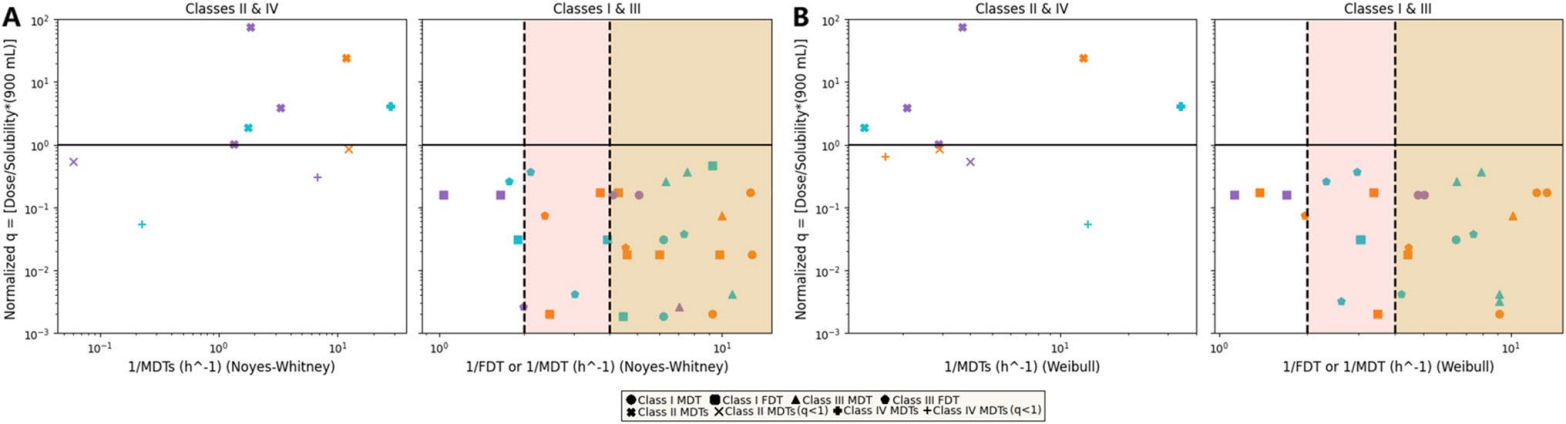
Temporal Biopharmaceutics Classification System (T-BCS) with scattered experimental data from the drugs listed in Table II. For both plots, the ordinate axis indicates the normalized dose/solubility ratio, *q*, in term of the volume of dissolution medium (900 mL), corresponding to the lowest solubility. On the left subplots, the horizontal time axis (h^−1^, logarithmic scale) corresponds to (1/M.D.T.s.) values for Class II and IV drugs whereas on the right subplots, the horizontal time axis (h^−1^, logarithmic scale) corresponds to (1/M.D.T.) or (1/F.D.T.) values for Class I and III drugs. The M.D.T., *τ*_d_ and M.D.T.s. values were calculated according to the expressions of Table I based on the Noyes-Whitney equation (Plot A) and Weibull function (Plot B). The shaded regions indicate the areas for drugs exhibiting rapid (30 min, pink shade at the 2 h^−1^ mark) or very rapid (15 min, beige shade marked at the 4 h^−1^ mark) dissolution in terms of the biowaiver’s criteria. Orange data points correspond to dissolution medium of pH 1.2, purple data points correspond to dissolution medium of pH 4.5 and blue data points correspond to dissolution medium of pH 6.8.

Once the F.D.T. and M.D.T. (for Class I and III drugs) and the M.D.T.s. (for Class II and IV) values (h) were estimated graphically as described above in the Methods section (Eq. 3), they were plotted with the normalized Dose/Solubility ratio, *q*, which was calculated for each drug product individually using Eq. 1. The dose that was utilized was the highest dose (mg) and the solubility *C*_s_ (mg/mL) corresponding to the three pH values (1.2, 4.5 and 6.8) that all the dissolution tests were carried out. For the volume of the dissolution medium, *V* (mL), we employed the actual volume of the medium that was used in the dissolution tests in the literature (900 mL). It is important to note that within the context of the biopharmaceutics classification system (BCS), the specified volume is set at 250 mL, aligning with the typical volume of gastrointestinal fluids. The calculation of the M.D.T. and M.D.T.s. value was feasible only for the dissolution curves that unequivocally attained a plateau. Despite our efforts to obtain M.D.T. and M.D.T.s. values for each of the pH levels across all drug products, this was not achievable in certain instances. Similarly, the estimation of the F.D.T. values for Class I and III drugs was not feasible in cases where the dissolution medium led to a plateau less than 100% dissolved. Plotting the 1/M.D.T. or 1/F.D.T. values with the normalized *q* for Class I and III drugs and the 1/M.D.T.s. values again with their corresponding *q* values, in the lines of the T-BCS frame, we obtain Fig. 3.

In a similar vein, we plotted the M.D.T., M.D.T.s. and *τ*_d_ that were obtained through the Noyes-Whitney and Weibull fittings with the corresponding normalized Dose/Solubility ratios, *q*, resulting in Fig. 3.

## Discussion

Regarding Fig. 2, theoretically, one would anticipate *q* values for Class I and III drugs to be less than 1 because their solubility in the pH range should enable them to fully dissolve the highest dose in the given volume (900 mL). In fact, all Class I and III drugs satisfy the inequality *q* < 1 while most of the data points lie beyond the 2 h^−1^ mark (> 80% of the total Class I and III data points) (less than 30 min-rapidly dissolved limit for biowaiver status granting), Fig. 2. In parallel, *q* values for Class II and IV drugs should exceed 1 due to their solubility limitations, resulting in a saturated solution at the end of the dissolution process. However, some observations deviated from this expectation, Fig. 2. In this vein, enclosed data points for Class II and IV drugs with *q* < 1 are noted. Black circles highlight drugs (ketoprofen and piroxicam) previously classified in Classes I and II of the BCS [35–37, 42, 57]. Red circle mark drug (amodiaquine hydrochloride) previously classified in Classes III and IV of the BCS [49, 57]. It should be noted that the classification in Fig. 2 relies on a fixed volume of 900 mL and reveals that less than 50% of the Class II and IV dataset (5 out of 12, accounting for 41.67%) is positioned below the threshold of *q* = 1 and almost all are positioned within the range of *q* = 0.1 to *q* = 1, as evident when considering the logarithmic scale, Fig. 2.

The exact location of the drug in the x-axis coupled with the *q* value quantifies the Class II or IV character within each T-BCS region, individual points are defined by their specific *q* and M.D.T./M.D.T.s. values. This implies that the positioning of these data points reflects the heterogeneous nature of compounds within the same class. When considering Class II and IV data points, it becomes crucial to account for two factors: the *q* value, which directly stems from the solubility of the compound, and the M.D.T.s. values, particularly their relative placement in relation to the borderline with the M.D.T. values. The precise coordinates of these data points serve as a quantitative measure that sheds light on the compound's behavior and the interplay between the two dissolution mechanisms. As elaborated before, it's important to note that under both *in vitro* and *in vivo* conditions, a single mechanism does not exclusively operate. The inadequacy of the reaction-limited mechanism is intricately linked to the complexity arising from the simultaneous involvement of multiple dissolution mechanisms in these scenarios.

As evident from Figs. 3A and 3B, the data points conform to the same pattern observed in Fig. 2. Most of the Class I and III drugs are positioned beyond the 2 h^−1^ threshold, indicating that they exhibit mean dissolution and finite dissolution times of less than 30 min, which is the time limit for rapidly dissolved drugs. Similarly, an equivalent number of data points pertaining to Class II and IV drugs are situated below the *q* = 1 line, as previously explained in the context of Fig. 3. When compared, Figs. 2, 3A and 3B show a slightly different data point distribution. Noteworthy data points in Figs. 2 and 3, beyond the 10 h^−1^ threshold and below the *q* = 1 threshold, belonging to either Class I/II or III/IV [27], exhibited release and dissolution profiles that are akin to drugs from Class I/III that reach % dissolved profiles > 85% in 30 min. In fact, similar dissolution profiles for ketoprofen piroxicam and amodiaquine hydrochloride in dissolution media 1.2 were reported [35–37, 42, 49]. Thus, analysis of the dissolution data revealed that, these drugs exhibit dissolution profiles exceeding 86% dissolved within approximately 45 min for piroxicam, less than 30 min for ketoprofen, and less than 60 min for amodiaquine hydrochloride. This underscores the substantial influence of their dual classification (Class I/II for ketoprofen, piroxicam and III/IV for amodiaquine hydrochloride) on their dissolution profiles, particularly evident in the case of ketoprofen.

**Fig. 4:**
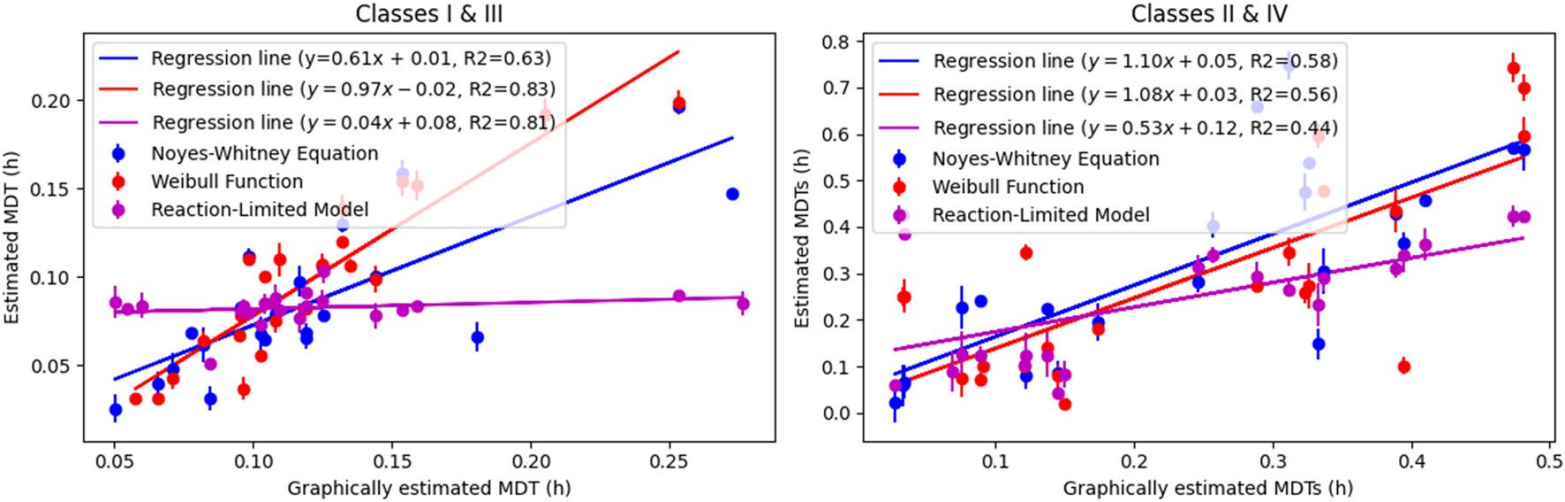
Temporal parameters correlation plot: The left and right subplots contain experimental data (h) for Class I/III and estimated values (h) using the Noyes-Whitney (blue), Weibull function (red), and reaction-limited model (purple), while the abscissa presents graphically calculated M.D.T. values (h). Each model’s color-coded trendline derived from linear regression analysis is also shown in the legends.

Our next goal was to explore potential relationships between the various M.D.T. and M.D.T.s. values, estimated using the Noyes-Whitney equation, the Weibull function, and the reaction-limited model of dissolution with those calculated from the graphical method, which simply relies on the experimental data using Eq. 2. To visualize and quantify the relationships among these three sets of models, we generated a correlation plot, Fig. 4; the inspection of the plots reveals that the Weibull function has the best performance since in both plots the slope of the regression lines is close to unity and the intercept is close to zero. The corresponding correlation coefficients, *R*^2^ are 0.83 for Class I/III drugs and 0.56 for Class II/IV drugs. These values are supportive for the correlation of the variables (parameters) analyzed if one considers the diversity of data in terms of inter-and intra-Class variation (both *q* > 1 and *q* < 1 Class II/IV drugs are included) and the longer (double) time span of the Class II/IV drugs’ data in comparison with the Class I/III drugs’ data. It seems that the Weibull function captures much better the dynamics of the dissolution process across all data analyzed since the fundamental differential equation (Eq. 9) describes a first-order process with a time dependent coefficient driving the dissolution rate. In fact, the analytical power of the Weibull function for the discernment of dissolution-release process in homogenous /heterogeneous media have been previously depicted in [15]. Regarding the Noyes Whitney model for both Classes I/III and II/IV drugs the intercept is close to zero, the slopes are 0.61 and 1.10 and the correlation coefficients are 0.63 and 0.58, respectively. These results show that the Noyes Whitney model has comparable performance with the Weibull model only for Classes II/IV drugs. This can be associated with recent findings, which indicate that soluble compounds follow the diffusion limited model, while sparingly soluble drugs follow the reaction limited model of dissolution [58, 59]. In the same vein, for the reaction-limited model, the most noticeable observation is the lack of correlation for Class I/III drugs, with a correlation coefficient of only 0.04.

Although the better performance of the Weibull model is perhaps not surprising given it provides a more "flexible" fit compared to Noyes Whitney, the poor performance of the reaction-limited model even for BCS II/IV drugs requires consideration in the light of the dissolution mechanisms operating under *in vitro* and *in vivo* conditions*.* After so many years of drug dissolution research, the prevailing dissolution mechanism relies on the diffusion layer model; however, there are many reports in literature which justify the reaction-limited dissolution model. For example, in a 2022 study, [60], Sleziona *et al*. discussed the particle dissolution behavior of a highly soluble and a sparingly soluble compound using a theoretical geometrical phase-field approach. They confirmed that the prevailing mechanism in the case of the highly soluble compound was indeed the diffusion layer model whereas the reaction limited, in their case surface-reaction limited, case was the prevailing model for the sparingly soluble compound. This theoretical work is related to two previously published studies [58, 61]. In the former study, carried out under hydronamically controlled *in vitro* conditions, the two mechansms seem to operate simultaneously. The latter study links the supersaturated phenomena, which are usually encountered with Class II and IV drugs with the reaction-limited model of drug dissolution. Overall, there has not been a single case where a compound follows only a single mechanism to the full extent under *in vitro* and *in vivo* conditions. It is obvious that easily dissolved drugs (like Class I and III drugs) have much shorter finite dissolution time values and more simple dissolution profiles in comparison with sparingly soluble drugs which have much longer M.D.T.s and more complex dissolution profiles (s-shaped). This means that when we attempt to correlate *in vitro* and *in vivo* results, it is much more difficult to predict Class II and Class IV behavior instead of the other two classes. Finally, it should be noted that all phenomena stated above are a function of the agitation rate which are drammatically different under *in vitro* and *in vivo* conditions. Based on all above,the poor performace of the reaction limited model of dissolution, using various drugs from different BCS classes under *in vitro* and *in vivo* conditiosn is a plausible result.

The mean and median estimates and their standard deviation and range, respectively, of the M.D.T., M.D.T.s. and *τ*_d_ parameters are listed in Table III for Class I/III and Class II/IV drugs. A comparison of mean with the median estimates reveals their similarity except for the M.D.T.s. graphical and Noyes Whitney estimates and to a lesser degree reaction limited estimates for Class II and IV drugs. For these three sets of results the median is more appropriate as a measure of the central tendency of the data. In all other cases, the mean describes the data adequately. It should be also noted that the Weibull function had the best performance in terms of statistical performance since for all parameters studied for Class I/III and II/IV drugs the mean estimates were associated with small standard deviations and the corresponding median values were very similar. Although the sample is small (see Table II), the magnitude of the parameters roughly follows the expected ranking M.D.T. < *τ*_d_ < M.D.T.s. It should be noted that the inequality M.D.T. < τ_d_ is reasonable since M.D.T. reflects the mean behaviour of solid particles in temrs of the time scale of the dissolution process while τ_d_ refers to the time for the completion of the disolution process of the solid drug particles. In this vein, the M.D.T. estimtes being 2–3 folds shorter than τ_d_ should not be used as metrics for the rapid or very rapid dissolving drug classification. Nevertheless, the M.D.T. and τ_d_ estimates are useful if contrasted with the F.A.T. estimates derived from the analysis of blood concentration time data [62] or the percent absorbed *versus* time plots [1] for the development of *in vitro in vivo* correlations.

**Table III:** Mean (± SD) and median (range) time parameters estimates using the graphical and modeling approaches for Class I/III and Class II/IV drugs listed in Table II

Model	Temporal parameter	Class I/III	Class II/IV
Graphical	*τ*_*d*_ (h)	0.28 (± 0.4)[mean] 0.33 (0.086–1.86)[median]	-
	M.D.T. (h)	0.13 (± 0.06)[mean] 0.109 (0.050–0.373)[median]	-
	M.D.T.s. (h)	-	2.3 (± 7) [mean] 0.32 (0.027–30) [median]
Noyes-Whitney	*τ*_*d*_ (h)	0.35 (± 0.2)[mean] 0.29 (0.084–0.97)[median]	-
	M.D.T. (h)	0.098 (± 0.05)[mean] 0.083 (0.026–0.24)[median]	-
	M.D.T.s. (h)	-	2.7 (± 6) [mean] 0.42 (0.018–24) [median]
Weibull	*τ*_*d*_ (h)	0.33 (± 0.16)[mean] 0.29 (0.084–0.88)[median]	-
	M.D.T. (h)	0.11 (± 0.05)[mean] 0.106 (0.031–0.21)[median]	-
	M.D.T.s. (h)	-	0.27 (± 0.2) [mean] 0.25 (0.018–0.74)[median]
Reaction-limited	*τ*_*d*_ (h)	-	-
	M.D.T. (h)	0.07917 (± 0.00882) [mean] 0.08093 (0.05143–0.10293) [median]	-
	M.D.T.s. (h)	-	0.46 (± 0.7)[mean] 0.29 (0.034–3.2)[median]

## Conclusions

Although BCS is fundamentally a qualitative system used for categorizing drugs based on their solubility and permeability characteristics, the T-BCS introduces a novel dimension by complementing and expanding a previously reported quantitative biopharmaceutical classification system [63]. This approach enables the establishment of correlations, the assessment of magnitudes of time dissolution parameters, and the comparison of different drugs, offering valuable insights into the classification of drugs within the BCS framework.
